# Regional aortic wall shear stress increases over time in patients with a bicuspid aortic valve

**DOI:** 10.1016/j.jocmr.2024.101070

**Published:** 2024-08-02

**Authors:** Savine C.S. Minderhoud, Aïmane Arrouby, Allard T. van den Hoven, Lidia R. Bons, Raluca G. Chelu, Isabella Kardys, Dimitris Rizopoulos, Suze-Anne Korteland, Annemien E. van den Bosch, Ricardo P.J. Budde, Jolien W. Roos-Hesselink, Jolanda J. Wentzel, Alexander Hirsch

**Affiliations:** a Department of Cardiology, Erasmus Medical Center, University Medical Center Rotterdam, Rotterdam, the Netherlands; b Department of Radiology and Nuclear Medicine, Erasmus Medical Center, University Medical Center Rotterdam, Rotterdam, the Netherlands; c Department of Biostatistics, Erasmus Medical Center, University Medical Center Rotterdam, Rotterdam, the Netherlands

**Keywords:** 4D flow CMR, Bicuspid aortic valve, Wall shear stress, Aortic diseases

## Abstract

**Background:**

Aortic wall shear stress (WSS) is a known predictor of ascending aortic growth in patients with a bicuspid aortic valve (BAV). The aim of this study was to study regional WSS and changes over time in BAV patients.

**Methods:**

BAV patients and age-matched healthy controls underwent four-dimensional (4D) flow cardiovascular magnetic resonance (CMR). Regional, peak systolic ascending aortic WSS, aortic valve function, aortic stiffness measures, and aortic dimensions were assessed. In BAV patients, 4D flow CMR was repeated after 3 years of follow-up and both at baseline and follow-up computed tomography angiography (CTA) were acquired. Aortic growth (volume increase of ≥5%) was measured on CTA. Regional WSS differences within patients’ aorta and WSS changes over time were analyzed using linear mixed-effect models and were associated with clinical parameters.

**Results:**

Thirty BAV patients (aged 34 years [interquartile range (IQR) 25–41]) were included in the follow-up analysis. Additionally, another 16 BAV patients and 32 healthy controls (aged 33 years [IQR 28–48]) were included for other regional analyses. Magnitude, axial, and circumferential WSS increased over time (all p < 0.001) irrespective of aortic growth. The percentage of regions exposed to a magnitude WSS >95th percentile of healthy controls increased from 21% (baseline 506/2400 regions) to 31% (follow-up 734/2400 regions) (p < 0.001). WSS angle, a measure of helicity near the aortic wall, decreased during follow-up. Magnitude WSS changes over time were associated with systolic blood pressure, peak aortic valve velocity, aortic valve regurgitation fraction, aortic stiffness indexes, and normalized flow displacement (all p < 0.05).

**Conclusion:**

An increase in regional WSS over time was observed in BAV patients, irrespective of aortic growth. The increasing WSSs, comprising a larger area of the aorta, warrant further research to investigate the possible predictive value for aortic dissection.

## Graphical abstract

**Figure:**
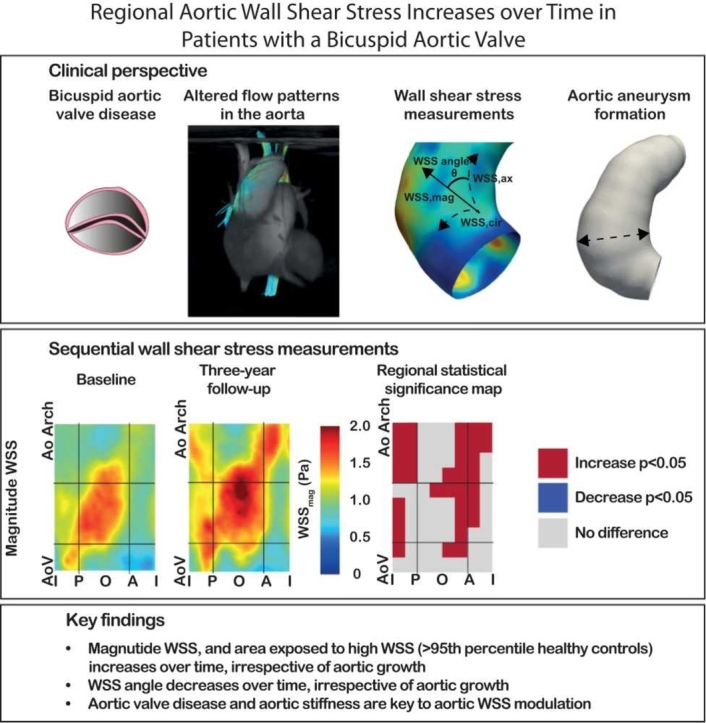

## 1 Introduction

Bicuspid aortic valve (BAV) patients are at risk of developing aortic dilation early in life and this has been attributed to genetic and hemodynamic factors [1]. Due to the altered valve morphology, abnormal flow patterns develop in the aorta with increased shearing of blood flow over the aortic wall. The local impact of flow on the aortic wall is assessed by measuring wall shear stress (WSS) as can be evaluated with four-dimensional (4D) flow cardiovascular magnetic resonance (CMR) and its magnitude and direction vary from region to region [2], [3], [4]. 4D flow-based WSS measurements tend to underestimate the true WSS compared to, e.g., WSS calculated via computational fluid dynamics (CFD) [5], [6]. However, 4D flow CMR provides the correct WSS distribution and is more accessible compared to CFD for daily patient care.

Through local mechanotransduction, WSS regulates vascular homeostasis and can initiate and progress arterial disease [7]. Previous research indicates that the increase and decrease of WSS over time, rather than the absolute levels of WSS, are critical to vascular homeostasis and remodeling, including changes in vessel wall diameter [8]. In BAV disease, regions with increased WSS correspond with the region with aortic wall degeneration [9]. In fact, the magnitude of the WSS, the direction of the WSS, the WSS angle, and the size of the area of the aortic wall exposed to elevated WSS are all three associated with the degree of aortic growth [4], [10], [11], indicating the potential value of WSS measurements in prediction the risk of aortic dissection in BAV patients.

Studies describing regional WSS changes over time, also in the context of changes in aortic size and peak aortic valve velocity [12], are missing. Given the low disease progression in the general BAV population [13], [14], in this study, 4D flow CMR was used to study regional WSS changes over time in high-risk BAV patients. Patients were considered high risk if at least moderate valvular aortic disease or aortic dilation was present [15], [16]. Furthermore, associations were studied of morphological and hemodynamic changes, such as aortic dilation, with regional WSS changes.

## 2 Materials and methods

### 2.1 Study population

High-risk patients with a BAV were prospectively included. Patients underwent 4D flow CMR imaging, echocardiography, and computed tomography angiography (CTA) on the same day at inclusion and again after 3 years of follow-up. Inclusion criteria were 1) peak aortic velocity >2.5 m/s, 2) aortic regurgitation ≥moderate, or 3) ascending aortic diameter ≥40 mm and/or aortic size index >2.1 cm/m^2^. Exclusion criteria were patients with syndromic aortic pathology, such as Turner syndrome, age <18 years, pregnancy, or contra-indications for administering contrast media. To evaluate the natural progression of the disease, patients who underwent aortic (valve) surgery during the follow-up period were excluded. For the comparison of regional WSS, healthy controls were recruited and age-matched at a group level. Inclusion criteria for healthy controls were being an adult (age ≥18 years) without a history of cardiovascular disease. There were no follow-up scans available of the healthy controls. The study was approved by the local ethics committee (MEC-2014-225 NL and MEC-2014-096 NL). All participants provided written informed consent.

### 2.2 Cardiovascular magnetic resonance

Imaging acquisition was performed using a 1.5T clinical MRI scanner (Discovery MR450 or SIGNA Artist, both GE Healthcare, Milwaukee, Wisconsin) using a 32-channel phased-array cardiac surface or anterior phased-array coil. The imaging protocol has been described before. In short, it consisted of black blood turbo spin echo images of the aorta, two-dimensional (2D) phase contrast images at aortic valve level, 2D phase contrast images in the ascending aorta at the level of the pulmonary bifurcation, steady-state free precession images at the level of pulmonary trunk, and 4D flow CMR of the entire thoracic aorta [4].

4D flow CMR was performed with an acquired resolution of 1.8 × 2.1 × 2.8 mm, a temporal resolution of 44–51 ms, 20 reconstructed phases per cardiac cycle, echo time 3.8–4.2 ms, repetition time 1.5–2.3 ms, and flip angle 15°. 4D flow CMR was acquired using retrospective cardiac gating during free breathing with respiratory motion compensation in the axial plane after administration of gadolinium-based contrast agent. The flow-encoding scheme was symmetric four-point, velocity encoding was set at 180 cm/s for healthy controls and at 250 cm/s for patients and velocity encoding was increased if necessary up to 550 cm/s [4].

### 2.3 Computed tomography

Acquisition was performed using a dual-source computed tomography (CT) (Somaton Force or Somatom Definition Flash, Siemens Healthineers, Forchheim, Germany). Retrospective ECG-gated spiral acquisition was applied. The systolic phase was selected and a reconstruction was made with a slide thickness of 1.0 mm and 0.6 mm overlap. A 65 mL bolus of iodinated contrast material (Iodixanol 320, Visipaque, GE Healthcare, Cork, Ireland) was administered and image acquisition was started using bolus tracking in the ascending aorta.

### 2.4 Image analysis

**Fig. 1:**
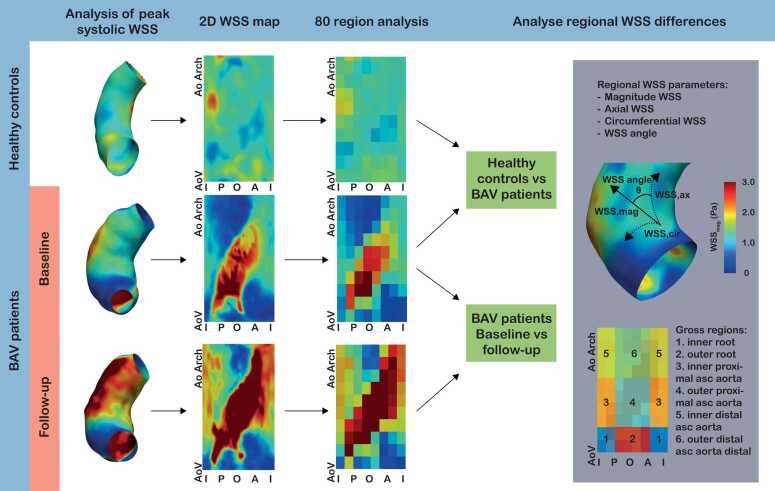
Graphical overview of the regional wall shear stress (WSS) analysis. *BAV* bicuspid aortic valve, *AoV* aortic valve, *AoArch* start of aortic arch, *I* inner, *P* posterior, *O* outer, *A* anterior.

Ascending aortic WSS on 4D flow CMR was analyzed using CAAS MR Solutions 5.1 (Pie Medical Imaging, Maastricht, the Netherlands) and the methodology has been described previously in detail [4]. In short, the aortic wall was automatically segmented by the software and was subsequently checked by the analyst and manually adapted where necessary*.* WSS was analyzed at the peak systolic phase, defined as the phase with the maximum flow (mL/s) in the ascending aorta, the phase before, and after this phase. Results of these three cardiac phases were averaged and analyzed in a regional manner (Fig. 1). The ascending aorta, between the aortic annulus and innominate artery, was divided into 10 parts longitudinally and 8 parts circumferentially (45°) resulting in 80 regions. These 80 regions were grouped according to their location in the ascending aorta into 6 gross regions: inner and outer aortic root, inner and outer proximal ascending aorta, inner and outer distal ascending aorta. The aortic root was defined as the first 20% of the ascending aortic length distal to the aortic valve, proximal ascending aorta was the following 20% to 60%, and distal ascending aorta was defined as the distal 60% to 100% of the length. Inner was defined as 180° at the inner curvature and outer as 180° around the outer curvature. Magnitude, axial, and circumferential WSS and the direction, the angle between magnitude and axial WSS, were analyzed separately (Fig. 1). WSS angle was calculated with the following formula:

$$\mathrm{WSS angle}\left({}^{\circ}\right)=\tan^{-1}\left(\frac{\mathrm{circumferential WSS}}{\mathrm{axial WSS}}\right)$$

To measure flow eccentricity, normalized displacement was measured 5 mm above the valve coaptation using Qflow 5.6 (Medis, Leiden, The Netherlands) as described before [17]. In short, the definition of flow displacement was the distance between the center of the lumen and the center of velocity of the forward flow, and was normalized to the lumen diameter. The center of velocity was calculated as the average position of lumen pixels weight by the velocity information. Normalized displacement was measured during systole by averaging the displacement across 10% of the phases adjacent to the peak systolic flow.

Aortic regurgitation fraction was quantified on phase contrast images at the aortic valve level after applying stationary phantom correction Qflow 8.1 (Medis, Leiden, The Netherlands) [18]. Aortic distensibility and pulse wave velocity were measured on steady-state free precession images at the level of pulmonary bifurcation and 2D phase contrast images in the ascending aorta at the level of the pulmonary bifurcation, using the methodology as described before [4]. Ascending aortic distensibility was calculated as follows:

$$\mathrm{ascending aortic distensibility}\left(mmHg^{-1}\right)=\;\frac{\mathrm{maximum area}\left({\mathrm{mm}}^{2}\right)-\mathrm{minimum area}\left({\mathrm{mm}}^{2}\right)\;}{\mathrm{minimum area}\left({\mathrm{mm}}^{2}\right)\times \mathrm{brachial pulse pressure}\left(mmHg\right)}$$

Pulse wave velocity was measured of the aortic arch on the slice positioned perpendicular to the ascending and descending aorta. Pulse wave velocity was defined as:

$$\mathrm{pulse}\;\;\mathrm{wave}\;\;\mathrm{velocity}\left(m/s\right)=\;\frac{\mathrm{ascending}-\mathrm{descending distance}\left(m\right)}{∆\;\;\mathrm{time}\left(s\right)}$$

The distance between the aortic measurements was measured on a sagittal angulated T1-weight black blood turbo spin echo images of the thoracic aorta. The time difference was calculated by drawing a line along the systolic upslope through the points at 20% and 80% of the maximum flow of the flow curve in ascending and descending aorta. The intersection point between this line (one of the ascending aortic flow curve and one of the descending aortic flow curve) and the x-axis was taken and the difference in time between the intersection points of ascending line and descending line was the difference in time. Aortic valve peak velocity was measured on echocardiography in BAV patients and on 4D flow CMR in healthy controls.

CTA was acquired on the same day as the CMR. The acquisition protocol, diameter, and volume measurements have been described earlier [4]. Using the double-oblique technique, in systole, diameters were measured of the aortic root from cusp-to-cusp, the sinotubular junction, and the widest portion of the ascending aorta. Aortic diameters were indexed to height. The level with the maximum growth was defined as the level at which the largest difference in millimeters was measured between baseline and follow-up. Aortic ascending volumes were measured on CTA using 3mensio software (Pie Medical Imaging, Maastricht, the Netherlands). Aortic growth was defined as volume growth ≥5% of the proximal ascending aorta (defined as the first 5 cm after the aortic valve) during follow-up [4].

### 2.5 Statistical analysis

Continuous variables are presented as mean with standard deviation or median with interquartile range (IQR). Categorical data are presented as frequencies and percentages. Differences in baseline characteristics and aortic measurements between patients and healthy controls were tested for significance using a Mann-Whitney test. Change between baseline and follow-up of all other factors, except WSS, was tested with paired samples Wilcoxon tests. For WSS, linear mixed models were used as explained below.

Linear mixed models with a random intercept for each patient were used to study regional WSS differences. The WSS parameters were modeled as dependent variables, whereas the study group (patients or healthy controls), valvular subtype, and timepoint (baseline or follow-up), respectively, were considered independent variables. We transformed the dependent WSS parameters using log2 when the residual plots showed deviation from normality. To account for spatial autocorrelation, a spatial Gaussian correlation structure was used in the models, based on the x-, y- and z-coordinates of regions (analyses within the aorta). Standardized x-, y- and z-coordinates of regions were used for comparisons between aortas (patients vs healthy controls, analyses per BAV subtype, baseline vs follow-up, growth vs no growth). The change in the number of regions exposed to high WSS values was tested with general linear mixed models, with the presence of increased WSS modeled as the dependent variable and valvular subtypes and timepoint (baseline or follow-up) as independent variables.

To estimate WSS changes over time, linear mixed models were created with regional changes of magnitude WSS or WSS angle over time as a dependent variable. In the fixed-effects part, baseline characteristics were entered as independent variables and random intercepts were used for each patient. To assess whether there is an effect modification of WSS changes over time by demographic parameters, aortic diameters, and valvular pathology, linear mixed models were created including time and each of these covariables, as well as their interaction terms as fixed factors. These models were adjusted for age and maximum aortic diameter. Random intercepts were used for each patient. Covariables with a p < 0.25 were entered in each multivariable model. Intra-observer repeatability of regional WSS measurements has been described in a previous study and was excellent (intraclass correlation coefficient = 0.92) [19].

Analyses have been performed in R Statistical Software version 4.1.0 (R Foundation for Statistical Computing, Vienna, Austria). Two-tailed p-values below 0.05 were considered statistically significant.

## 3 Results

### 3.1 Study population

Forty-six BAV patients (30 males (65%), median age 33 years (IQR 24–41)) were included (Table 1). Of these 46 patients, in 30 patients (median age 34 years (IQR 25–41)), 4D flow CMR was repeated after 3 years of follow-up (1096 days (IQR 1080–1106, Table 2). Follow-up 4D flow CMR was missing in the remaining 16 patients for various reasons: 3 patients underwent aortic (valve) surgery, 3 patients declined to participate in the follow-up visit, and in 10 patients CMR was not acquired due to technical or logistical reasons. Of the 30 patients with follow-up, aortic volume of the proximal ascending aorta grew significantly over 3 years (baseline 49 (IQR (44–63) to 53 cm^3^ (IQR 45–65), p = 0.004) (Table 3). The maximum aortic diameter growth occurred most frequently in the ascending aorta. In 14 of 30 (47%) patients, the maximum aortic diameters were equal at two or three levels.

**Table 1:** Baseline characteristics.

	Healthy controls	Bicuspid aortic valve patients	p-value
	(n = 32)	(n = 46)	
Age (years)	33 (28–48)	33 (24–41)	0.390
Male	15 (47%)	30 (65%)	0.110
Weight (kg)	71 (65–83)	76 (69–85)	0.141
Height (cm)	178 (172–184)	183 (172–188)	0.141
Body mass index (kg/m^2^)	23 (22–24)	24 (22–25)	0.496
Heart rate (beats/min)	59 (54–66)	68 (57–76)	0.005
Systolic blood pressure (mmHg)	110 (103–118)	121 (112–131)	<0.001
Diastolic blood pressure (mmHg)	70 (65–76)	76 (70–84)	<0.001
Aortic valve morphology			
BAV Sievers type 0	-	11 (24%)	
BAV Sievers type 1 LR	-	23 (50%)	
BAV Sievers type 1 RN	-	6 (13%)	
BAV Sievers type 2	-	6 (13%)	
Peak aortic valve velocity (m/s)	1.1 (1.0–1.2)*	2.6 (1.9–3.4)†	<0.001
Aortic valve stenosis (≥moderate)	-	18 (39%)†	
Forward flow (mL/beat)	93 (83–111)*	113 (96–128)*	0.003
Aortic regurgitation fraction (%)	0 (0–1)*	5 (2–14)*	<0.001
Aortic root diameter (mm)	32 (30–34)*	40 (35–44)‡	<0.001
Aortic root diameter (mm/m)	18 (17–19)*	22 (20–24)‡	<0.001
Sinotubular junction diameter (mm)	27 (25–30)*	35 (31–38)‡	<0.001
Sinotubular junction diameter (mm/m)	15 (15–16)*	19 (16–21)‡	<0.001
Ascending aorta diameter (mm)	28 (27–30)*	42 (36–46)‡	<0.001
Ascending aorta diameter (mm/m)	16 (15–17)*	23 (20–25)‡	<0.001

Values are presented as numbers (percentages) or median (interquartile range).

*BAV* bicuspid aortic valve, *LR* left-right coronary cusp, *RN* right-non coronary cusp.

* CMR-derived.

† Echo-derived.

‡ CT-derived.

**Table 2:** Characteristics in BAV patients with follow-up at baseline and during follow-up (n = 30).

	Baseline	Follow-up	p-value
Age (years)	34 (25–41)	37 (28–43)	<0.001
Male	23 (77%)	-	-
Weight (kg)	79 (72–85)	80 (74–86)	0.132
Height (cm)	184 (178–191)	-	-
Heart rate (beats/min)	66 (58–74)	66 (57–77)	0.509
Systolic blood pressure (mmHg)	121 (111–131)	116 (112–123)	0.482
Diastolic blood pressure (mmHg)	80 (74–83)	74 (66–78)	0.052
Aortic valve morphology			
BAV Sievers type 0	7 (23%)	-	-
BAV Sievers type 1 LR	16 (53%)	-	-
BAV Sievers type 1 RN	3 (10%)	-	-
BAV Sievers type 2	4 (13%)	-	-
Peak aortic valve velocity (m/s)*	2.6 (1.9–3.4)	2.9 (1.9–3.4)	0.434
Forward flow (mL/beat)†	113 (97–128)	124 (107–137)	0.181
Aortic regurgitation fraction (%)†	5 (2–11)	3 (2–16)	0.289
Aortic root diameter (mm)‡	40 (36–44)	41 (37–44)	0.145
Aortic root diameter (mm/m)‡	22 (20–24)	22 (20–24)	0.211
Sinotubular junction diameter (mm)‡	35 (31–39)	35 (31–39)	0.295
Sinotubular junction diameter (mm/m)‡	19 (17–21)	19 (17–21)	0.250
Ascending aorta diameter (mm)‡	44 (37–46)	44 (37–47)	0.050
Ascending aorta diameter (mm/m)‡	23 (20–25)	24 (21–26)	0.162

Values are presented as numbers (percentages) or median (interquartile range).

*BAV* bicuspid aortic valve, *LR* left-right coronary cusp, *RN* right-non coronary cusp.

* Echo-derived.

† CMR-derived.

‡ CT-derived.

**Table 3:** Aortic diameters and volumes in BAV patients with follow-up.

	BAV patients (n = 30)	BAV patients with aortic growth (n = 14)	BAV patients without aortic growth (n = 16)
Aortic diameter at level with maximum growth baseline (mm)	41 (34–45)	39 (34–46)	42 (36–45)
Aortic diameter at level with maximum growth follow-up (mm)	42 (35–46)	41 (35–46)	42 (37–46)
p-value baseline vs FU*	<0.001	0.003	0.098
Proximal ascending aorta volume baseline (cm^3^)	49 (44–63)	47 (45–58)	51 (43–66)
Proximal ascending aorta volume follow-up (cm^3^)	53 (45–65)	53 (47–63)	50 (44–65)
p-value baseline vs FU*	0.004	0.001	0.438

*FU* follow-up, *BAV* bicuspid aortic valve.

* Comparing baseline with follow-up using Wilcoxon paired one sample test.

Fourteen (47%) patients had aortic volume growth of at least 5%. Characteristics of the BAV patients were compared to characteristics of healthy controls (Table 1).

### 3.2 Regional WSS analysis

**Fig. 2:**
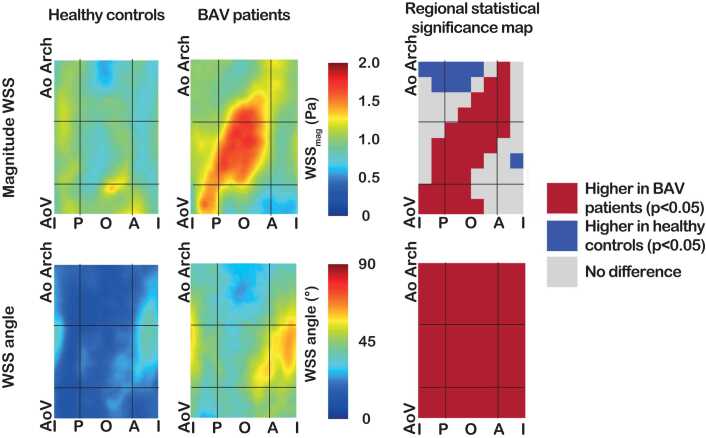
Average maps of baseline magnitude wall shear stress (WSS) and WSS angle in healthy controls (n = 32) versus bicuspid aortic valve (BAV) patients (n = 46) and significance maps showing regional differences between BAV patients and healthy controls. Black lines indicate the division of the ascending aorta into the gross regions. *AoV* aortic valve, *AoArch* start of aortic arch, *I* inner, *P* posterior, *O* outer, *A* anterior.

Fig. 2 summarizes the regional comparison of magnitude WSS and WSS angle between BAV patients and healthy controls. In BAV patients, magnitude, axial, and circumferential WSS were the highest in the outer proximal ascending aorta, irrespective of BAV morphology (Fig. 2 and Supplementary Table 1). Both in patients and healthy controls, WSS angle peaked in the inner proximal ascending aorta, the region with the most helical flow at the aortic wall, not corresponding to regions with highest magnitude WSS in BAV patients (Fig. 2). Circumferential WSS and WSS angle were higher in BAV patients compared to healthy controls in all regions, indicating there is more helical flow at the aortic wall in the entire ascending aorta (Fig. 2, Supplementary Table 1). The outer proximal ascending aorta was the region with the most profound differences between BAV patients and controls (magnitude WSS, circumferential WSS, and WSS angle all higher, all p < 0.001, Supplementary Table 1). Regional WSS was also analyzed per valvular subtype and is described in Supplementary Fig. 1. There was a tendency toward increasing WSS values and more helicity in higher Sievers subtypes.

### 3.3 Longitudinal analysis of hemodynamic factors

**Fig. 3:**
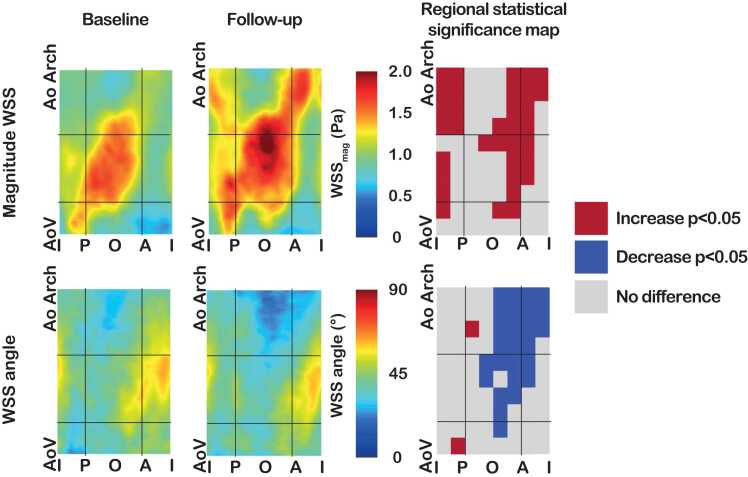
Average maps of baseline versus 3-year follow-up of magnitude wall shear stress (WSS) and WSS angle in bicuspid aortic valve patients and significance maps showing regional differences between baseline and follow-up. Black lines indicate the division of the ascending aorta into the gross regions. AoV: aortic valve, AoArch: start of aortic arch, I: inner, P: posterior, O: outer, A: anterior.

**Fig. 4:**
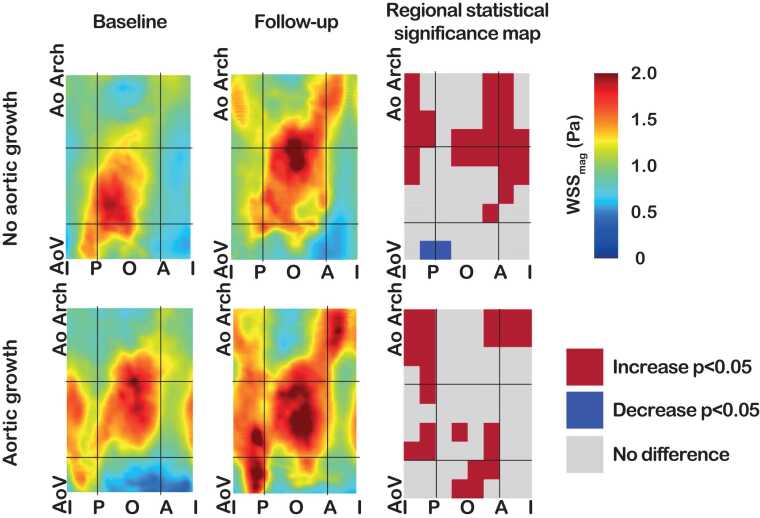
Average maps of baseline versus 3-year follow-up of magnitude wall shear stress (WSS) in bicuspid aortic valve patients with and without aortic growth and significance maps showing regional differences between baseline and follow-up. Black lines indicate the division of the ascending aorta into the gross regions. *AoV* aortic valve, *AoArch* start of aortic arch, *I* inner, *P* posterior, *O* outer, *A* anterior.

Table 4 shows the change in WSS parameters during a 3-year follow-up. Assessing the entire ascending aorta, the magnitude, axial, and circumferential WSS increased significantly over 3 years (all p < 0.001) with a relative change of all components of >10%, while the WSS angle decreased (baseline 42° [IQR 30–56] vs follow-up 40° [IQR 27–54], β − 1.9 (95% confidence interval (CI) −2.7, −1.1), p < 0.001). Magnitude WSS significantly increased in all regions of the proximal and distal ascending aorta (all, p < 0.001), but especially in the region around the hotspot in the outer proximal ascending aorta (Fig. 3 and Supplementary Table 2). Of all 80 regions in all patients, the number of regions with a magnitude WSS higher than 1.45 Pa (95th percentile of healthy controls) increased from 506/2400 (21.1%) regions to 734/2400 (30.6%) regions (β 0.09 (95% CI 0.07, 0.11), p < 0.001). Axial WSS increased in the proximal and distal ascending aorta (all, p < 0.001). Circumferential WSS increased in all regions (all, p < 0.001), except for outer distal ascending aorta (Supplementary Table 2). The WSS angle decreased in the outer proximal ascending aorta and the distal ascending aorta, suggesting decreasing helicity in these regions, while the WSS angle increased in the inner aortic root (Fig. 4 and Supplementary Table 2).

**Table 4:** Regional wall shear stress changes over time stratified according to aortic growth at 3-year follow-up.

	BAV patients (n = 30)	BAV patients with aortic growth (n = 14)	BAV patients without aortic growth (n = 16)	β (95% CI)*	p-value*
Number of regions	2400	1120	1280		
Magnitude WSS baseline (Pa)	0.91 (0.62–1.34)	0.94 (0.66–1.41)	0.87 (0.59–1.28)	0.07 (−0.30, 0.43)	0.701
Magnitude WSS follow-up (Pa)	1.07 (0.75–1.62)	1.21 (0.86–1.86)	0.98 (0.71–1.43)	0.28 (−0.11, 0.67)	0.151
Change over time (Pa)	0.12 (−0.08 to 0.46)	0.18 (−0.05 to 0.60)	0.09 (−0.12 to 0.35)	0.20 (−0.07, 0.48)	0.134
β (95% CI)†^,^‡	0.27 (0.23–0.31)	0.30 (0.26, 0.34)	0.19 (0.14, 0.24)	-	-
p-value baseline vs FU†	<0.001	<0.001	<0.001	-	-
Axial WSS baseline (Pa)	0.57 (0.36–0.95)	0.56 (0.35–0.88)	0.58 (0.36–1.03)	−0.11 (−0.51, 0.28)	0.567
Axial WSS follow-up (Pa)	0.72 (0.45–1.5)	0.74 (0.48–1.27)	0.70 (0.44–1.06)	0.14 (−0.25, 0.54)	0.458
Change over time (Pa)	0.10 (−0.08 to 0.37)	0.16 (−0.05 to 0.50)	0.07 (−0.12 to 0.26)	0.21 (0.02, 0.41)	0.033
β (95% CI)†,‡	0.31 (0.26–0.36)	0.36 (0.31, 0.42)	0.21 (0.14, 0.28)	-	-
p-value baseline vs FU†	<0.001	<0.001	<0.001	-	-
Circumferential WSS baseline (Pa)	0.52 (0.34–0.79)	0.61 (0.37–0.92)	0.47 (0.32–0.69)	0.33 (−0.03, 0.68)	0.068
Circumferential WSS follow-up (Pa)	0.62 (0.37–0.98)	0.76 (0.47–1.17)	0.51 (0.32–0.83)	0.47 (0.02, 0.92)	0.042
Change over time (Pa)	0.06 (−0.10 to 0.30)	0.07 (−0.11 to 0.35)	0.05 (−0.10 to 0.24)	0.06 (−0.12, 0.23)	0.528
β (95% CI)†^,^‡	0.21 (0.16–0.25)	0.22 (0.17, 0.28)	0.15 (0.09, 0.21)	-	-
p-value baseline vs FU†	<0.001	<0.001	<0.001	-	-
WSS angle baseline (°)	42 (30–56)	45 (33–59)	38 (26–52)	6.9 (2.1, 11.8)	0.007
WSS angle follow-up (°)	40 (27–54)	43 (30–56)	36 (25–51)	5.2 (−0.6, 11.0)	0.077
Change over time (°)	−2 (−11 to 8)	−3 (−13 to 8)	−1 (−10 to 8)	−1.7 (−4.8, 1.5)	0.295
β (95% CI)†^,^§	−1.9 (−2.7, −1.1)	−2.9 (−4.2, −1.7)	−1.2 (−2.2, −0.1)	-	-
p-value baseline vs FU†	<0.001	<0.001	0.031	-	-

Values are presented as numbers or median (interquartile range). Linear mixed-effect models are created with the WSS parameter as dependent variable and timepoint (baseline or follow-up) as independent variable. Models have a random slope per patient and a spatial Gaussian correlation structure.

*BAV* bicuspid aortic valve*, FU* follow-up*, WSS* wall shear stress, *CI* confidence interval*.*

* Comparing patients with and without aortic growth.

† Comparing baseline with follow-up.

‡ Results are presented as the mean difference with 95% confidence interval (CI) of the WSS expressed as 2log Pa.

§ Results are presented as the mean difference with 95% confidence interval (CI) of the WSS expressed as °.

Both in patients with and without aortic growth, magnitude, axial, and circumferential WSS increased over time (all, p < 0.001, Fig. 4, Table 4). The WSS angle at baseline was higher in patients with aortic growth (aortic growth 45° [33–59] vs no aortic growth 38° [IQR 26–52], β 6.9 (95% CI 2.1, 11.8), p = 0.007), and the circumferential WSS at follow-up (aortic growth 0.76 (0.47–1.17) vs no aortic growth 0.51 (0.32–0.83), β 0.47 (95% CI 0.02, 0.92), p = 0.042). The WSS angle decreased both in patients with and without aortic growth.

Also, when including only BAV patients with no or mild aortic stenosis, magnitude, axial, and circumferential WSS increased over time (all, p < 0.001, Supplementary Table 3). WSS angle decreased during follow-up from 38° [IQR 26–52] at baseline to 35° [IQR 23–50] (β − 2.3 (95% CI −3.4, −1.3), p < 0.001). In BAV patients with Sievers type 0 (n = 7), magnitude WSS increased and WSS angle decreased during follow-up, while in BAV patients with Sievers type 1 LR (n = 16), magnitude, axial, and circumferential WSS increased over time. Comparing patients with BAV Sievers type 0 with Sievers type 1 LR, the magnitude WSS increased over time in both groups. Axial and circumferential WSS increased only significantly in patients with Sievers type 1 LR, while the WSS angle only significantly decreased in patients with Sievers type 0. WSS angle decreased more in patients with BAV Sievers type 0 than in patients with Sievers type 1 LR.

During follow-up, blood pressure, heart rate, stroke volume, aorta valve velocity, aortic regurgitation fraction, and normalized flow displacement remained stable. Aortic distensibility decreased from 4.1 mmHg^−1^ [IQR 2.6–7.3] to 3.6 mmHg^−1^ [IQR 2.0–5.4] (β − 0.9 (95% CI −2.6, −0.1, p = 0.031) and pulse wave velocity increased from 5.0 m/s [IQR 3.9–5.8] to 6.0 m/s [IQR 4.6–7.4] (β 1.2 (95% CI 0.3, 2.1), p = 0.006), suggesting that the aorta stiffens.

### 3.4 Factors predicting and associated with WSS change over time

Baseline peak aortic valve velocity and regurgitation fraction were independently associated with an increase in magnitude WSS over time. When baseline aortic valve velocity was 1 m/s higher, this resulted in 0.166 Pa more increase of magnitude WSS during follow-up, while a regurgitation fraction of 1% lower at baseline resulted in a 0.018 Pa more increase of the magnitude WSS. Normalized flow displacement was the only predictor of an increasing WSS angle, meaning patients with more normalized flow displacement at baseline developed more helical flow during follow-up and their WSS angle increased (Supplementary Table 4). Change of magnitude WSS was not associated with baseline maximal aortic diameter.

Systolic blood pressure, peak aortic valve velocity, normalized flow displacement, aortic regurgitation fraction, aortic pulse wave velocity, and distensibility were all, independently of age and maximum aortic diameter, associated with differences in temporal change of the magnitude WSS (Supplementary Table 5). Consequently, patients with increasing peak aortic velocity and normalized flow displacement have more WSS increase, whereas an increasing severity of aortic valve regurgitation and stiffening of the aorta results in less WSS increase over time (all p-values for interaction with time p < 0.05). The increase in WSS was not dependent on the valvular subtype (data not shown). With regard to WSS angle, increasing normalized flow displacement, peak aortic valve velocity, and maximum aortic ascending diameter were associated with an increase in WSS angle and an increasing aortic distensibility with a decrease in WSS angle (Supplemental Table 5).

## 4 Discussion

This study found 1) that abnormal bicuspid valve morphology impacted the local hemodynamics at the outer proximal ascending aorta the most, while the entire ascending aortic wall of BAV patients was exposed to higher WSS angles compared to healthy controls and the magnitude of the WSS angle depends on the valve subtype, 2) regional magnitude, axial, and circumferential WSS all increased over 3 years, irrespective of aortic dilation, and 3) aortic valve stenosis, aortic valve regurgitation, and aortic stiffness were the most important contributors to changes in WSS over time.

The regional aortic wall analyses in this young patient cohort showed an increase in all WSS components over time, especially in the proximal and distal ascending aorta. Larger regions in the aorta became exposed to high WSSs, which are associated with aortic dilation [10]. In our study, the increase of WSS and the decrease of WSS angle occurred both in patients with and without aortic dilation, and interestingly, the change of magnitude WSS was not associated with baseline aortic diameters. Previous studies showed that WSS is a factor driving aortic growth and remodeling, and this study shows WSS remains a driver, irrespective of the degree of aortic dilation and even while the aorta widens [4], [10], [11]. Previous retrospective follow-up studies have reported conflicting results demonstrating either reduction or stabilization of WSS over time despite aortic dilation [10], [20], [21]. Despite the selection of high-risk BAV patients, peak aortic valve velocities in these previous studies were comparable to peak aortic valve velocities in our study. However, all previous studies investigating WSS changes over time measured averaged global WSS values and none looked at regional differences. In this study, we noticed clear regional WSS differences, with the highest WSS values being measured in the outer proximal ascending aorta. WSS is thought to be a regional biomarker that acts through mechanotransduction. Regional WSS changes over time might be masked by measuring average WSS change and this could potentially explain the differences with previous studies [20], [21], [22]. In a recent study in low-risk BAV patients, the area exposed to elevated WSS and the averaged peak systolic WSS remained stable over a 5-year period [23]. Based on our study results, the development of valvular disease is important for the WSS changes. However, in the study by Maroun et al., it is unclear how valvular disease develops during follow-up and how this relates to the WSS changes, which makes a comparison to our results hard.

This is the first study showing an increase in regional WSS parameters over the years, irrespective of aortic dilation, while WSS was expected to decrease as a reaction to aortic widening. Physiologically, mechanotransduction initiates a process that can lead to lowering of WSS in case of increased WSS. The aortas of BAV patients are distinct on a molecular level from aortas of tricuspid aortic valve patients. Possibly BAV patients also have a mechanotransduction defect, which could lead to an adapted and less effective response to WSS alterations, and prevent WSS lowering [24].

Increased WSS over time might be best explained by the evolution of valvular disease as aortic valve stenosis accelerated the increase of WSS over time, whereas aortic valve regurgitation and aortic stiffening protected the aorta against an increase of WSS. This partly extends previous research showing patients with stiffer aortas have a decreased endothelial response and are potentially less able to change their aortic WSS [25].

Regardless of aortic valve morphology, our results consistently show that the outer wall of the proximal ascending aorta is exposed to the highest shearing forces, confirming previous findings [12]. In correspondence with a histological study, regions exposed to high WSS were foremost collected from the outer proximal ascending aorta, and these regions were associated with the location of extracellular dysregulation and elastic fiber degeneration [3]. Regarding aortic dissection, limited data are available about the exact location of the entry tear in patients with BAV. It remains to be determined whether the outer proximal ascending aorta is also where an aortic dissection typically starts in BAV patients.

BAV patients with higher Sievers classifications are associated with high WSS, larger aortic area exposed to increased WSS, and more helical flow, in line with a small cohort study [26]. Previously, it has been shown that the presence and number of raphes are associated with the risk of aortic valve stenosis and regurgitation, however, not yet with aortopathy [27]. The present study suggests that patients with more raphes have higher WSS values and are potentially more prone to aortic dilation. This finding should first be confirmed in larger studies but suggests that more frequent monitoring might be appropriate in patients with higher Sievers subtypes for aortopathy.

## 5 Limitations

The low spatial resolution and high segmentation offset could underestimate the true WSS values [28]. In this study, the spatial resolution and segmentation offset were similar to the 4D flow scans in patients and healthy controls and also of the scan at the baseline and follow-up moment, therefore, comparisons within our population could not have resulted in bias. Given the tendency to underestimate WSS with 4D flow, absolute WSS values should not directly be compared between acquisition sites and other methods to determine the WSS, such as CFD [3], [4]. There was no follow-up available in the healthy controls, this precludes to study the WSS changes over time in age-matched healthy controls. Only high-risk BAV patients were included, therefore, this study cannot determine whether WSSs also increase in low-risk BAV patients. Furthermore, patients requiring aortic (valve) surgery were excluded, hereby we excluded the most affected patients from our study. This may have influenced our results. Patient’s individual loading conditions (blood pressure, heart rate, and fluid balance) may have been different between baseline and follow-up; however, no systematic bias is expected. Finally, given the small population of patients, we were unable to perform a detailed regional analysis of WSS changes over time per valve subtype.

## 6 Conclusions

We demonstrated that the entire ascending aortic wall of BAV patients is exposed to a higher circumferential WSS and larger WSS angle compared to healthy controls and WSS levels and magnitude of WSS angle at the aortic wall are dependent on aortic valve subtype. Given that WSS levels are associated with aortic dilation, patients with a higher Sievers’ classification might need closer surveillance. From our follow-up WSS analyses, aortic wall remodeling and homeostasis seem to fail, as WSS levels increase even further, irrespective of aortic dilation. Over time, the area of the aorta exposed to high WSS increases, possibly making the aorta more prone to further dilation. The degree of aortic valvular disease and aortic stiffness indices in particular seem responsible for the WSS changes.

## Funding

This research was funded by a grant from the 10.13039/501100002996Dutch Heart Foundation (grant number: 2013T093) and the Thorax Foundation.

## Author contributions

S.C.S.M., J.J.W., and A.H. designed the study. S.C.S.M., R.G.C., R.P.J.B., and A.H. performed the clinical CMR scanning. S.C.S.M. performed all WSS and aortic volume measurements. A.A. performed the flow eccentricity measurements and L.R.B. the aortic diameter measurements. S.C.S., I.K., and D.R. performed the statistical data analyses. S.C.S.M., J.J.W., and A.H. drafted the manuscript. All authors read and approved the final manuscript.

## Ethics approval and consent

The study was approved by the local ethics committee (MEC-2014-225 NL and MEC-2014-096 NL). All participants provided written informed consent.

## Consent for publication

Not applicable.

## Declaration of competing interests

The authors declare the following financial interests/personal relationships which may be considered as potential competing interests: Alexander Hirsch reports financial support was provided by Thorax Foundation. Jolien Roos-Hesselink reports financial support was provided by Dutch Heart Foundation. Alexander Hirsch reports a relationship with GE Healthcare that includes consulting or advisory, funding grants, and speaking and lecture fees. Alexander Hirsch reports a relationship with Bayer AG that includes speaking and lecture fees. Alexander Hirsch reports a relationship with Medis Medical Imaging Systems BV that includes consulting or advisory. Alexander Hirsch reports a relationship with Cardialysis BV that includes board membership. Alexander Hirsch is the associate editor of the Journal of Cardiovascular Magnetic Resonance. The other authors declare that they have no known competing financial interests or personal relationships that could have appeared to influence the work reported in this paper.

## Data Availability

The datasets used and/or analyzed during the current study are available from the corresponding author on reasonable request.

## References

[1] Verma S., Siu S.C. (2014). Aortic dilatation in patients with bicuspid aortic valve. N Engl J Med.

[2] Hope M.D., Hope T.A., Meadows A.K., Ordovas K.G., Urbania T.H., Alley M.T. (2010). Bicuspid aortic valve: four-dimensional MR evaluation of ascending aortic systolic flow patterns. Radiology.

[3] Guzzardi D.G., Barker A.J., Van Ooij P., Malaisrie S.C., Puthumana J.J., Belke D.D. (2015). Valve-related hemodynamics mediate human bicuspid aortopathy: insights from wall shear stress mapping. J Am Coll Cardiol.

[4] Minderhoud S.C.S., Roos-Hesselink J.W., Chelu R.G., Bons L.R., Van Den Hoven A.T., Korteland S.A. (2022). Wall shear stress angle is associated with aortic growth in bicuspid aortic valve patients. Eur Heart J Cardiovasc Imaging.

[5] Szajer J., Ho-Shon K. (2018). A comparison of 4D flow MRI-derived wall shear stress with computational fluid dynamics methods for intracranial aneurysms and carotid bifurcations—a review. Magn Reson Imaging.

[6] Cibis M., Potters W.V., Gijsen F.J.H., Marquering H., vanBavel E., van der Steen A.F.W. (2014). Wall shear stress calculations based on 3D cine phase contrast MRI and computational fluid dynamics: a comparison study in healthy carotid arteries. NMR Biomed.

[7] Humphrey J.D., Milewicz D.M., Tellides G., Schwartz M.A. (2014). Dysfunctional mechanosensing in aneurysms. Science (1979).

[8] Chiu J.J., Chien S. (2011). Effects of disturbed flow on vascular endothelium: pathophysiological basis and clinical perspectives. Physiol Rev.

[9] Guzzardi D.G., Barker A.J., van Ooij P., Malaisrie S.C., Puthumana J.J., Belke D.D. (2015). Valve-related hemodynamics mediate human bicuspid aortopathy: insights from wall shear stress mapping. J Am Coll Cardiol.

[10] Soulat G., Scott M.B., Allen B.D., Avery R., Bonow R.O., Malaisrie S.C. (2022). Association of regional wall shear stress and progressive ascending aorta dilation in bicuspid aortic valve. JACC Cardiovasc Imaging.

[11] Guala A., Dux-Santoy L., Teixido-Tura G., Ruiz-Muñoz A., Galian-Gay L., Servato M.L. (2022). Wall shear stress predicts aortic dilation in patients with bicuspid aortic valve. JACC Cardiovasc Imaging.

[12] van Ooij P., Markl M., Collins J.D., Carr J.C., Rigsby C., Bonow R.O. (2017). Aortic valve stenosis alters expression of regional aortic wall shear stress: new insights from a 4-dimensional flow magnetic resonance imaging study of 571 subjects. J Am Heart Assoc.

[13] Detaint D., Michelena H.I., Nkomo V.T., Vahanian A., Jondeau G., Sarano M.E. (2014). Aortic dilatation patterns and rates in adults with bicuspid aortic valves: a comparative study with Marfan syndrome and degenerative aortopathy. Heart.

[14] Della Corte A., Bancone C., Buonocore M., Dialetto G., Covino F.E., Manduca S. (2013). Pattern of ascending aortic dimensions predicts the growth rate of the aorta in patients with bicuspid aortic valve. JACC Cardiovasc Imaging.

[15] Tzemos N., Therrien J., Yip J., Thanassoulis G., Tremblay S., Jamorski M.T. (2008). Outcomes in adults with bicuspid aortic valves. JAMA.

[16] Michelena H.I., Khanna A.D., Mahoney D., Margaryan E., Topilsky Y., Suri R.M. (2011). Incidence of aortic complications in patients with bicuspid aortic valves. JAMA.

[17] Sigovan M., Hope M.D., Dyverfeldt P., Saloner D. (2011). Comparison of four-dimensional flow parameters for quantification of flow eccentricity in the ascending aorta. J Magn Reson Imaging.

[18] Minderhoud S.C.S., van der Velde N., Wentzel J.J., van der Geest R.J., Attrach M., Wielopolski P.A. (2020). The clinical impact of phase offset errors and different correction methods in cardiovascular magnetic resonance phase contrast imaging: a multi-scanner study. J Cardiovasc Magn Reson.

[19] Minderhoud S.C.S., Fletcher A.J., MacNaught G., Cadet S., Korteland S.A., Kardys I. (2022). Vascular biomechanics and molecular disease activity in the thoracic aorta: a novel imaging method. Eur Heart J Cardiovasc Imaging.

[20] Rose M.J., Rigsby C.K., Berhane H., Bollache E., Jarvis K., Barker A.J. (2019). 4-D flow MRI aortic 3-D hemodynamics and wall shear stress remain stable over short-term follow-up in pediatric and young adult patients with bicuspid aortic valve. Pediatr Radiol.

[21] Rahman O., Scott M., Bollache E., Suwa K., Collins J., Carr J. (2019). Interval changes in aortic peak velocity and wall shear stress in patients with bicuspid aortic valve disease. Int J Cardiovasc Imaging.

[22] O’Rourke M.F., Nichols W.W. (2005). Aortic diameter, aortic stiffness, and wave reflection increase with age and isolated systolic hypertension. Hypertension.

[23] Maroun A., Scott M.B., Catania R., Berhane H., Jarvis K., Allen B.D. (2024). Multiyear interval changes in aortic wall shear stress in patients with bicuspid aortic valve assessed by 4D flow MRI. J Magn Reson Imaging.

[24] van de Pol V., Kurakula K., DeRuiter M.C., Goumans M.J. (2017). Thoracic aortic aneurysm development in patients with bicuspid aortic valve: what is the role of endothelial cells?. Front Physiol.

[25] McEniery C.M., Wallace S., MacKenzie I.S., McDonnell B., Newby D.E., Cockcroft J.R. (2006). Endothelial function is associated with pulse pressure, pulse wave velocity, and augmentation index in healthy humans. Hypertension.

[26] Stephens E.H., Hope T.A., Kari F.A., Kvitting J.P.E., Liang D.H., Herfkens R.J. (2015). Greater asymmetric wall shear stress in Sievers’ type 1/LR compared with 0/LAT bicuspid aortic valves after valve-sparing aortic root replacement. J Thorac Cardiovasc Surg.

[27] Kong W.K.F., Delgado V., Poh K.K., Regeer M. v, Ng A.C.T., McCormack L. (2017). Prognostic implications of raphe in bicuspid aortic valve anatomy. JAMA Cardiol.

[28] Potters W.V., Van Ooij P., Marquering H., Van Bavel E., Nederveen A.J. (2015). Volumetric arterial wall shear stress calculation based on cine phase contrast MRI. J Magn Reson Imaging.

