# Supplementary material for: Regional aortic wall shear stress increases over time in patients with a bicuspid aortic valve
Source: J Cardiovasc Magn Reson. 2024 Aug 2;26(2):101070. doi: 10.1016/j.jocmr.2024.101070 (PMC11417319; doi:10.1016/j.jocmr.2024.101070)
Supplement: Supplementary file 1 — Supplementary material [file mmc1.docx]

## **Supplementary data**

| **Supplementary Table 1 Regional wall shear stress analysis in healthy controls versus bicuspid aortic valve patients** | | | | | | | |
| --- | --- | --- | --- | --- | --- | --- | --- |
|  | Entire aorta | Inner root | Outer root | Inner proximal ascending aorta | Outer proximal ascending aorta | Inner distal ascending aorta | Outer distal ascending aorta |
| Number of regions controls | 2560 | 256 | 256 | 512 | 512 | 512 | 512 |
| Number of regions patients | 3680 | 368 | 368 | 736 | 736 | 736 | 736 |
|  |  |  |  |  |  |  |  |
| Magnitude WSS controls (Pa) | 0.87 (0.68-1.08) | 1.00 (0.77-1.18) | 1.00 (0.77-1.21) | 0.89 (0.68-1.10) | 0.85 (0.69-1.02) | 0.88 (0.69-1.10) | 0.77 (0.59-1.00) |
| Magnitude WSS patients (Pa) | 0.96 (0.66-1.40) | 0.78 (0.51-1.24) | 0.79 (0.47-1.19) | 0.94 (0.69-1.28) | 1.29 (0.89-1.85) | 0.92 (0.65-1.24) | 0.93 (0.65-1.42) |
| β (95% CI) ǁ* | -0.12 (-0.32, 0.07) | 0.25 (0.02, 0.48) | 0.36 (0.24, 0.53) | -0.15 (-0.35, 0.05) | -0.47 (-0.70, -0.24) | -0.09 (-0.31, 0.12) | -0.32 (-0.56, -0.08) |
| p-value ǁ | 0.211 | 0.035 | 0.004 | 0.140 | <0.001 | 0.386 | 0.011 |
|  |  |  |  |  |  |  |  |
| Axial WSS controls (Pa) | 0.87 (0.68-1.09) | 0.93 (0.72-1.19) | 0.93 (0.72-1.13) | 0.79 (0.57-0.98) | 0.78 (0.62-0.92) | 0.79 (0.61-0.99) | 0.73 (0.56-0.96) |
| Axial WSS patients (Pa) | 0.62 (0.38-1.00) | 0.54 (0.32-0.90) | 0.51 (0.28-0.90) | 0.50 (0.34-0.75) | 0.76 (0.49-1.11) | 0.58 (0.38-0.93) | 0.71 (0.49-1.12) |
| β (95% CI) ǁ* | 0.34 (0.14, 0.55) | 0.71 (0.46, 0.96) | 0.85 (0.57-1.12) | 0.46 (0.26, 0.66) | 0.04 (-0.25, 0.32) | 0.35 (0.10, 0.61) | 0.00 (-0.27, 0.27) |
| p-value ǁ | 0.001 | <0.001 | <0.001 | <0.001 | 0.802 | 0.007 | 0.988 |
|  |  |  |  |  |  |  |  |
| Circumferential WSS controls (Pa) | 0.25 (0.17-0.35) | 0.24 (0.18-0.31) | 0.28 (0.20-0.40) | 0.29 (0.20-0.44) | 0.24 (0.16-0.34) | 0.28 (0.19-0.38) | 0.18 (0.13-0.25) |
| Circumferential WSS patients (Pa) | 0.54 (0.35-0.83) | 0.41 (0.28-0.65) | 0.44 (0.28-0.65) | 0.65 (0.45-0.92) | 0.76 (0.49-1.11) | 0.52 (0.37-0.77) | 0.41 (0.27-0.67) |
| β (95% CI) ǁ* | -1.09 (-1.29, -0.89) | -0.98 (-1.22, -0.74) | -0.64 (-0.90, -0.38) | -1.15 (-1.39, 0.91) | -1.58 (-1.84, -1.32) | -0.92 (-1.16, -0.68) | -1.25 (-1.51, -1.00) |
| p-value ǁ | <0.001 | <0.001 | <0.001 | <0.001 | <0.001 | <0.001 | <0.001 |
|  |  |  |  |  |  |  |  |
| WSS angle controls (°) | 16 (12-24) | 15 (11-19) | 18 (13-23) | 21 (14-33) | 16 (11-24) | 19 (14-27) | 13 (11-18) |
| WSS angle patients (°) | 41 (28-55) | 40 (30-51) | 42 (30-52) | 52 (39-65) | 42 (29-57) | 40 (29-56) | 30 (19-42) |
| β (95% CI) ǁ# | -22 (-25, -19) | -25 (-28, -21) | -23 (-27, -19) | -26 (-29, -23) | -24 (-29, -20) | -20 (-24, -16) | -18 (-21, -14) |
| p-value ǁ | <0.001 | <0.001 | <0.001 | <0.001 | <0.001 | <0.001 | <0.001 |
| Values are presented as median (interquartile range). Linear mixed effect models are created in the WSS parameter as dependent variable and study group (healthy control or patient) as independent variable. ǁ comparing healthy controls with BAV patients. * Results are presented as the mean difference with 95% confidence interval (CI) of the WSS expressed as 2log Pa.  # Results are presented as the mean difference with 95% confidence interval (CI) of the WSS expressed as °. Models have a random slope per patient and a spatial Gaussian correlation structure. WSS = wall shear stress | | | | | | | |

**WSS analysis per valvular subtype**

Regional WSS was also analysed per valvular subtype and findings are visualised in Supplementary Figure 1. There was a tendency towards more helicity in the higher Sievers subtypes. Interestingly, in these regions where the WSS parameters peaked (outer proximal ascending aorta for magnitude WSS and circumferential WSS, inner proximal ascending aorta for WSS angle), a tendency was observed towards increasing WSS values and helicity in the higher Sievers subtypes.


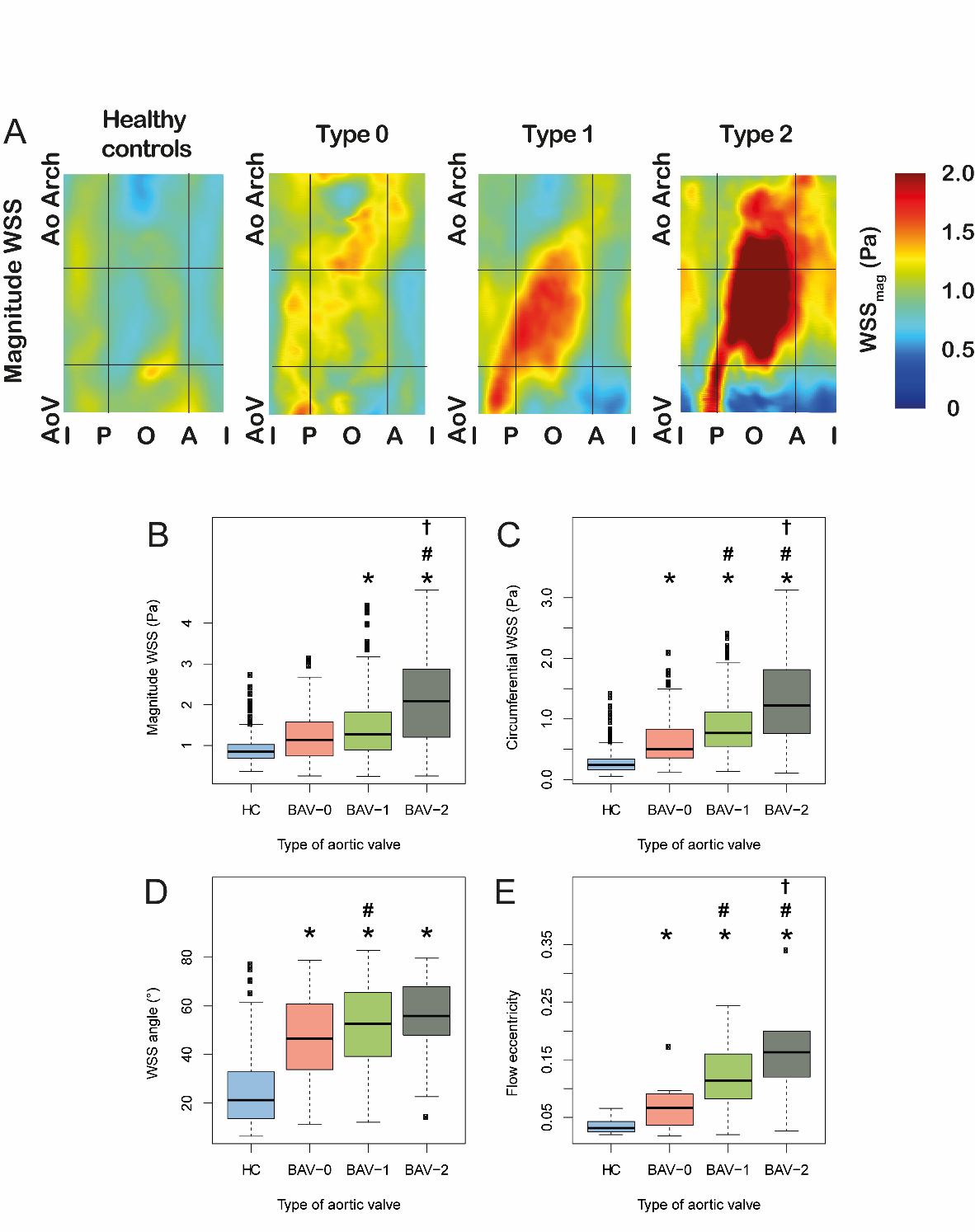


### **Supplementary Figure 1**: A) Average maps of baseline magnitude wall shear stress (WSS) per bicuspid aortic valve (BAV) subtype, B) magnitude WSS in outer proximal ascending aorta per aortic valve subtype, C) circumferential WSS in outer proximal ascending aorta per aortic valve subtype, D) WSS angle in inner proximal ascending aorta per aortic valve subtype and E) flow eccentricity per aortic valve subtype. Black lines in maps of panel A indicate the division of the ascending aorta into the gross regions; *p<0.05 compared to HC, # p<0.05 compared to BAV-0, † p<0.05 compared to BAV-1; AoV indicates aortic valve, AoArch start of aortic arch, I inner, P posterior, O outer and A anterior, HC healthy controls, BAV bicuspid aortic valve, BAV-0 BAV type 0, BAV-1 BAV type 1 and BAV-2 BAV type 2.

| **Supplementary Table 2 regional wall shear stress analysis of baseline versus follow-up in bicuspid aortic valve patients** | | | | | | | |
| --- | --- | --- | --- | --- | --- | --- | --- |
|  | Entire aorta | Inner root | Outer root | Inner proximal ascending aorta | Outer proximal ascending aorta | Inner distal ascending aorta | Outer distal ascending aorta |
| Number of regions | 2400 | 240 | 240 | 480 | 480 | 480 | 480 |
|  |  |  |  |  |  |  |  |
| Magnitude WSS baseline (Pa) | 0.91 (0.62-1.34) | 0.75 (048-1.15) | 0.77 (0.46-1.22) | 0.89 (0.64-1.23) | 1.23 (0.84-1.79) | 0.84 (0.61-1.19) | 0.87 (0.60-1.29) |
| Magnitude WSS follow-up (Pa) | 1.07 (0.75-1.62) | 0.75 (0.50-1.24) | 0.87 (0.60-1.33) | 1.08 (0.77-1.53) | 1.33 (0.96-2.21) | 1.08 (0.83-1.58) | 1.05 (0.72-1.62) |
| Change over time (Pa) | 0.12 (-0.08-0.46) | 0.01 (-0.18-0.21) | 0.08 (-0.12-0.42) | 0.11 (-0.06-0.37) | 0.13 (-0.10-0.52) | 0.22 (0.00-0.53) | 0.14 (-0.09-0.50) |
| β (95% CI) ǁ* | 0.27 (0.23-0.31) | 0.14 (0.00, 0.28) | 0.16 (0.03, 0.28) | 0.10 (0.06, 0.15) | 0.24 (0.18, 0.31) | 0.41 (0.35, 0.46) | 0.28 (0.21, 0.36) |
| p-value baseline vs FUǁ | <0.001 | 0.061 | 0.013 | <0.001 | <0.001 | <0.001 | <0.001 |
|  |  |  |  |  |  |  |  |
| Axial WSS baseline (Pa) | 0.57 (0.36-0.95) | 0.51 (0.30-0.87) | 0.49 (0.27-0.92) | 0.48 (0.32-0.76) | 0.79 (0.46-1.33) | 0.50 (0.35-0.81) | 0.65 (0.43-0.98) |
| Axial WSS follow-up (Pa) | 0.72 (0.45-1.5) | 0.55 (0.31-0.93) | 0.58 (0.36-0.99) | 0.60 (0.40-0.92) | 0.94 (0.60-1.60) | 0.75 (0.49-1.18) | 0.83 (0.54-1.34) |
| Change over time (Pa) | 0.10 (-0.08-0.37) | -0.01 (-0.15-0.17) | 0.04 (-0.13-0.28) | 0.07 (-0.08-0.24) | 0.13 (-0.08-0.46) | 0.18 (0.00-0.44) | 0.13 (-0.07-0.46) |
| β (95% CI) ǁ* | 0.31 (0.26-0.36) | 0.06 (-0.11, 0.22) | 0.12 (-0.03, 0.27) | 0.15 (0.07, 0.23) | 0.28 (0.20, 0.36) | 0.53 (0.44, 0.62) | 0.39 (0.30-0.48) |
| p-value baseline vs FU ǁ | <0.001 | 0.508 | 0.114 | <0.001 | <0.001 | <0.001 | <0.001 |
|  |  |  |  |  |  |  |  |
| Circumferential WSS baseline (Pa) | 0.52 (0.34-0.79) | 0.39 (0.28-0.60) | 0.42 (0.28-0.63) | 0.59 (0.41-0.84) | 0.74 (0.48-1.05) | 0.52 (0.36-0.75) | 0.43 (0.28-0.69) |
| Circumferential WSS follow-up (Pa) | 0.62 (0.37-0.98) | 0.44 (0.29-0.72) | 0.48 (0.33-0.75) | 0.74 (0.50-1.06) | 0.88 (0.55-1.26) | 0.65 (0.41-0.95) | 0.46 (0.25-0.81) |
| Change over time (Pa) | 0.06 (-0.10-0.30) | 0.04 (-0.09-0.18) | 0.07 (-0.09-0.28) | 0.07 (-0.06-0.31) | 0.06 (-0.16-0.35) | 0.07 (-0.09-0.34) | 0.03 (-0.14-0.25) |
| β (95% CI) ǁ* | 0.21 (0.16-0.25) | 0.24 (0.10, 0.38) | 0.25 (0.12, 0.37) | 0.16 (0.10, 0.22) | 0.20 (0.12, 0.28) | 0.25 (0.18, 0.32) | 0.07 (-0.02, 0.16) |
| p-value baseline vs FU ǁ | <0.001 | <0.001 | <0.001 | <0.001 | <0.001 | <0.001 | 0.147 |
|  |  |  |  |  |  |  |  |
| WSS angle baseline (°) | 42 (30-56) | 39 (29-50) | 41 (30-51) | 51 (37-65) | 44 (29-57) | 43 (32-58) | 33 (23-45) |
| WSS angle follow-up (°) | 40 (27-54) | 42 (33-53) | 41 (32-50) | 50 (39-63) | 41 (28-54) | 36 (25-54) | 27 (18-39) |
| Change over time (°) | -2 (-11-8) | 2 (-6-12) | 2 (-8-10) | 0 (-8-9) | -2 (-12-7) | -4 (-15-6) | -3 (-14-5) |
| β (95% CI) ǁ # | -1.9 (-2.7, -1.1) | 2.6 (0.4, 4.8) | 0.8 (-1.4, 3.0) | 0.0 (-1.4, 1.5) | -2.0 (-3.8, -0.3) | -5.1 (-6.8, -3.4) | -4.7 (-6.2, -3.2) |
| p-value baseline vs FU ǁ | <0.001 | 0.019 | 0.465 | 0.972 | 0.025 | <0.001 | <0.001 |
| Values are presented as numbers or median (interquartile range). Linear mixed effect models are created in the WSS parameter as dependent variable and timepoint (baseline or follow-up) as independent variable. Models have a random slope per patient and a spatial Gaussian correlation structure. ǁ comparing baseline with follow-up. * Results are presented as the mean difference with 95% confidence interval (CI) of the WSS expressed as 2log Pa. # Results are presented as the mean difference with 95% confidence interval (CI) of the WSS expressed as °. FU = follow-up, WSS = wall shear stress | | | | | | | |

| **Supplementary Table 3 Regional wall shear stress changes over time per subgroup at 3-year follow-up** | | | | | | | | |
| --- | --- | --- | --- | --- | --- | --- | --- | --- |
|  | **BAV patients**  **(n=30)** |  | **BAV patients with ≤ mild aortic stenosis**  **(n=18)** |  | **BAV type 0**  **(n=7)** | **BAV type 1 LR**  **(n=16)** | **β (95% CI) †** | **P-value †** |
| Number of regions | 2400 |  | 1440 |  | 560 | 1280 |  |  |
|  |  |  |  |  |  |  |  |  |
| Magnitude WSS baseline (Pa) | 0.91 (0.62-1.34) |  | 0.82 (0.57-1.23) |  | 0.96 (0.61-1.46) | 0.86 (0.60-1.24) | 0.10 (-0.37, 0.57) | 0.685 |
| Magnitude WSS follow-up (Pa) | 1.07 (0.75-1.62) |  | 0.96 (0.68-1.06) |  | 1.11 (0.69-1.81) | 1.01 (0.74-1.47) | 0.13 (-0.39, 0.65) | 0.630 |
| Change over time (Pa) | 0.12 (-0.08-0.46) |  | 0.08 (-0.09-0.33) |  | 0.05 (-0.13-0.44) | 0.15 (-0.04-0.40) | 0.07 (-0.28, 0.42) | 0.702 |
| β (95% CI) ǁ* | 0.27 (0.23-0.31) |  | 0.22 (0.17, 0.26) |  | 0.23 (0.15, 0.31) | 0.26 (0.20, 0.31) | - | - |
| P-value baseline vs FU ǁ | <0.001 |  | <0.001 |  | <0.001 | <0.001 | - | - |
|  |  |  |  |  |  |  |  |  |
| Axial WSS baseline (Pa) | 0.57 (0.36-0.95) |  | 0.55 (0.34-0.98) |  | 0.64 (0.36-1.19) | 0.56 (0.35-0.90) | 0.13 (-0.40, 0.67) | 0.623 |
| Axial WSS follow-up (Pa) | 0.72 (0.45-1.5) |  | 0.70 (0.42-1.06) |  | 0.88 (0.48-1.49) | 0.70 (0.43-1.02) | 0.29 (-0.23, 0.80) | 0.281 |
| Change over time (Pa) | 0.10 (-0.08-0.37) |  | 0.08 (-0.09-0.30) |  | 0.09 (-0.13-0.44) | 0.10 (-0.06-0.32) | 0.10 (-0.17, 0.37) | 0.474 |
| β (95% CI) ǁ* | 0.31 (0.26-0.36) |  | 0.27 (0.20, 0.33) |  | -0.00 (-0.05, 0.05) | 0.27 (0.20, 0.34) | - | - |
| P-value baseline vs FU ǁ | <0.001 |  | <0.001 |  | 0.967 | <0.001 | - | - |
|  |  |  |  |  |  |  |  |  |
| Circumferential WSS baseline (Pa) | 0.52 (0.34-0.79) |  | 0.46 (0.31-0.66) |  | 0.50 (0.34-0.72) | 0.49 (0.32-0.76) | -0.01 (-0.46, 0.44) | 0.980 |
| Circumferential WSS follow-up (Pa) | 0.62 (0.37-0.98) |  | 0.49 (0.31-0.79) |  | 0.52 (0.32-0.84) | 0.58 (0.35-0.95) | -0.10 (-0.71, 0.50) | 0.736 |
| Change over time (Pa) | 0.06 (-0.10-0.30) |  | 0.03 (-0.10-0.20) |  | -0.01 (-0.15-0.19) | 0.08 (-0.06-0.28) | -0.02 (-0.13, 0.09) | 0.856 |
| β (95% CI) ǁ* | 0.21 (0.16-0.25) |  | 0.13 (0.08, 0.19) |  | 0.08 (-0.00, 0.17) | 0.22 (0.16, 0.28) | - | - |
| P-value baseline vs FU ǁ | <0.001 |  | <0.001 |  | 0.071 | <0.001 | - | - |
|  |  |  |  |  |  |  |  |  |
| WSS angle baseline (°) | 42 (30-56) |  | 38 (26-52) |  | 38 (25-52) | 41 (29-55) | -2.2 (-9.0, 4.6) | 0.524 |
| WSS angle follow-up (°) | 40 (27-54) |  | 35 (23-50) |  | 33 (21-46) | 40 (27-55) | -6.0 (-13.1, 1.1) | 0.106 |
| Change over time (°) | -2 (-11-8) |  | -2 (-10-6) |  | -4 (-13-4) | -1 (-10-8) | -3.9 (-7.6, -0.2) | 0.046 |
| β (95% CI) ǁ # | -1.9 (-2.7, -1.1) |  | -2.3 (-3.4, -1.3) |  | -4.4 (-6.0, -2.9) | -1.0 (-2.2, 0.1) | - | - |
| P-value baseline vs FU ǁ | <0.001 |  | <0.001 |  | <0.001 | 0.072 | - | - |
| Values are presented as numbers or median (interquartile range). Linear mixed effect models are created in the WSS parameter as dependent variable and timepoint (baseline or follow-up) as independent variable. Models have a random slope per patient and a spatial Gaussian correlation structure. ǁ comparing baseline with follow-up. * Results are presented as the mean difference with 95% confidence interval (CI) of the WSS expressed as 2log Pa. # Results are presented as the mean difference with 95% confidence interval (CI) of the WSS expressed as °. † comparing patients with BAV 0 and with BAV type 1 LR. BAV = bicuspid aortic valve, FU = follow-up, WSS = wall shear stress | | | | | | | | |

| **Supplementary Table 4 baseline parameters associated with change of wall shear stress parameters over time in bicuspid aortic valve patients** | | | | |
| --- | --- | --- | --- | --- |
|  | **Delta magnitude WSS (Pa)** | | | |
|  | **Univariable analysis** | | **Multivariable analysis** | |
|  | **β (95% CI)** | **p-value** | **β (95% CI)** | **p-value** |
| **Age (years)** | 0.001 (-0.010 to 0.012) | 0.891 |  |  |
| **Systolic blood pressure (mmHg)** | -0.002 (-0.012 to 0.008) | 0.754 |  |  |
| **Diastolic blood pressure (mmHg)** | 0.003 (-0.011 to 0.018) | 0.641 |  |  |
| **Peak aortic valve velocity (m/s)** | 0.166 (0.024 to 0.309) | 0.022 | 0.163 (0.040 to 0.286) | 0.010 |
| **Normalized flow displacement** | 0.960 (-1.140 to 3.060) | 0.370 |  |  |
| **Forward flow per beat (mL/beat)** | 0.000 (-0.004 to 0.005) | 0.847 |  |  |
| **Aortic regurgitation fraction (%)** | -0.018 (-0.033 to -0.004) | 0.015 | -0.018 (-0.031 to -0.005) | 0.006 |
| **Aortic pulse wave velocity (m/s)** | 0.016 (-0.098 to 0.130) | 0.789 |  |  |
| **Aortic distensibility (mmHg^-1^)** | 0.004 (-0.026 to 0.034) | 0.791 |  |  |
| **Maximum ascending aortic diameter (mm)** | -0.010 (-0.030 to 0.010) | 0.306 |  |  |
|  | **Delta WSS angle (°)** | | | |
|  | **Univariable analysis** | | **Multivariable analysis** | |
|  | **β (95% CI)** | **p-value** | **β (95% CI)** | **p-value** |
| **Age (years)** | -0.003 (-0.131 to 0.137) | 0.968 |  |  |
| **Systolic blood pressure (mmHg)** | -0.026 (-0.141 to 0.089) | 0.655 |  |  |
| **Diastolic blood pressure (mmHg)** | 0.063 (-0.098 to 0.225) | 0.442 |  |  |
| **Peak aortic valve velocity (m/s)** | 1.169 (-0.543 to 2.882) | 0.181 |  |  |
| **Normalized flow displacement** | 30.27 (9.690 to 50.85) | 0.004 | 30.27 (9.690 to 50.85) | 0.004 |
| **Forward flow per beat (mL/beat)** | 0.000 (-0.050 to 0.051) | 0.985 |  |  |
| **Aortic regurgitation fraction (%)** | -0.078 (-0.263 to 0.107) | 0.409 |  |  |
| **Aortic pulse wave velocity (m/s)** | -0.218 (-1.533 to 1.097) | 0.745 |  |  |
| **Aortic distensibility (mmHg^-1^)** | -0.153 (-0.465 to 0.158) | 0.335 |  |  |
| **Maximum ascending aortic diameter (mm)** | 0.097 (-0.123 to 0.317) | 0.387 |  |  |
| β coefficient and p-values are from linear mixed models with a random slope and a spatial correlation structure. β coefficient means for example that patients with one unit higher peak aortic valve velocity at baseline the magnitude WSS increases with 0.166 Pa more over three years follow-up. WSS = wall shear stress; β = beta; CI = confidence interval | | | | |

| **Supplementary Table 5 conditional growth models of wall shear stress parameters over time in bicuspid aortic valve patients** | | | | | | | |  |
| --- | --- | --- | --- | --- | --- | --- | --- | --- |
|  | **Magnitude WSS (Pa)** | | | **WSS angle (°)** | | | | |
|  | **β (95% CI)** | | **p-value for interaction with time** | | **β (95% CI)** | | **p-value for interaction with time** | |
| **Age (years)** | 0.003 (0.000 to 0.006) | 0.118 | | 0.026 (-0.047 to 0.099) | | 0.490 | | |
| **Systolic blood pressure (mmHg)** | -0.004 (-0.007 to -0.001) | 0.018 | | -0.035 (-0.108 to 0.038) | | 0.347 | | |
| **Peak aortic valve velocity (m/s)** | 0.170 (0.124 to 0.213) | <0.001 | | 1.296 (0.341 to 2.251) | | 0.008 | | |
| **Normalized flow displacement** | 2.486 (1.729 to 3.243) | <0.001 | | 40.11 (24.26 to 55.95) | | <0.001 | | |
| **Forward flow per beat (mL/beat)** | 0.001 (0.000 to 0.002) | 0.196 | | -0.011 (-0.038 to 0.016) | | 0.422 | | |
| **Aortic regurgitation fraction (%)** | -0.019 (-0.024 to -0.015) | <0.001 | | -0.055 (-0.139 to 0.028) | | 0.194 | | |
| **Aortic pulse wave velocity (m/s)** | -0.093 (-0.133 to -0.054) | <0.001 | | -0.159 (-0.989 to 0.672) | | 0.708 | | |
| **Aortic distensibility (mmHg^-1^)** | 0.019 (0.002 to 0.037) | 0.027 | | -0.343 (-0.674 to -0.012) | | 0.042 | | |
| **Maximum ascending aortic diameter (mm)** | 0.000 (-0.006 to 0.006) | 0.933 | | 0.140 (0.012 to 0.267) | | 0.031 | | |
| Conditional growth models are linear mixed effect models with in the fixed effects an interaction term between time and the variable (e.g. peak aortic valve velocity) adjusted for age and maximum ascending aortic diameter and with a random slope and a spatial Gaussian correlation structure. This model also adjusts for changes in the fixed parameter (e.g. peak aortic valve velocity) over time. β coefficient means for example that patients with one unit higher of peak aortic valve velocity, given that the peak aortic valve velocity does not change over time, the magnitude WSS will increase with 0.170 Pa more over three years; WSS = wall shear stress, CI = confidence interval; β = beta | | | | | | | |  |
